# Supplementary figures and images for: Analysis of prognostic genes in the tumor microenvironment of lung adenocarcinoma
Source: PeerJ. 2020 Jul 23;8:e9530. doi: 10.7717/peerj.9530 (PMC7382940; doi:10.7717/peerj.9530)

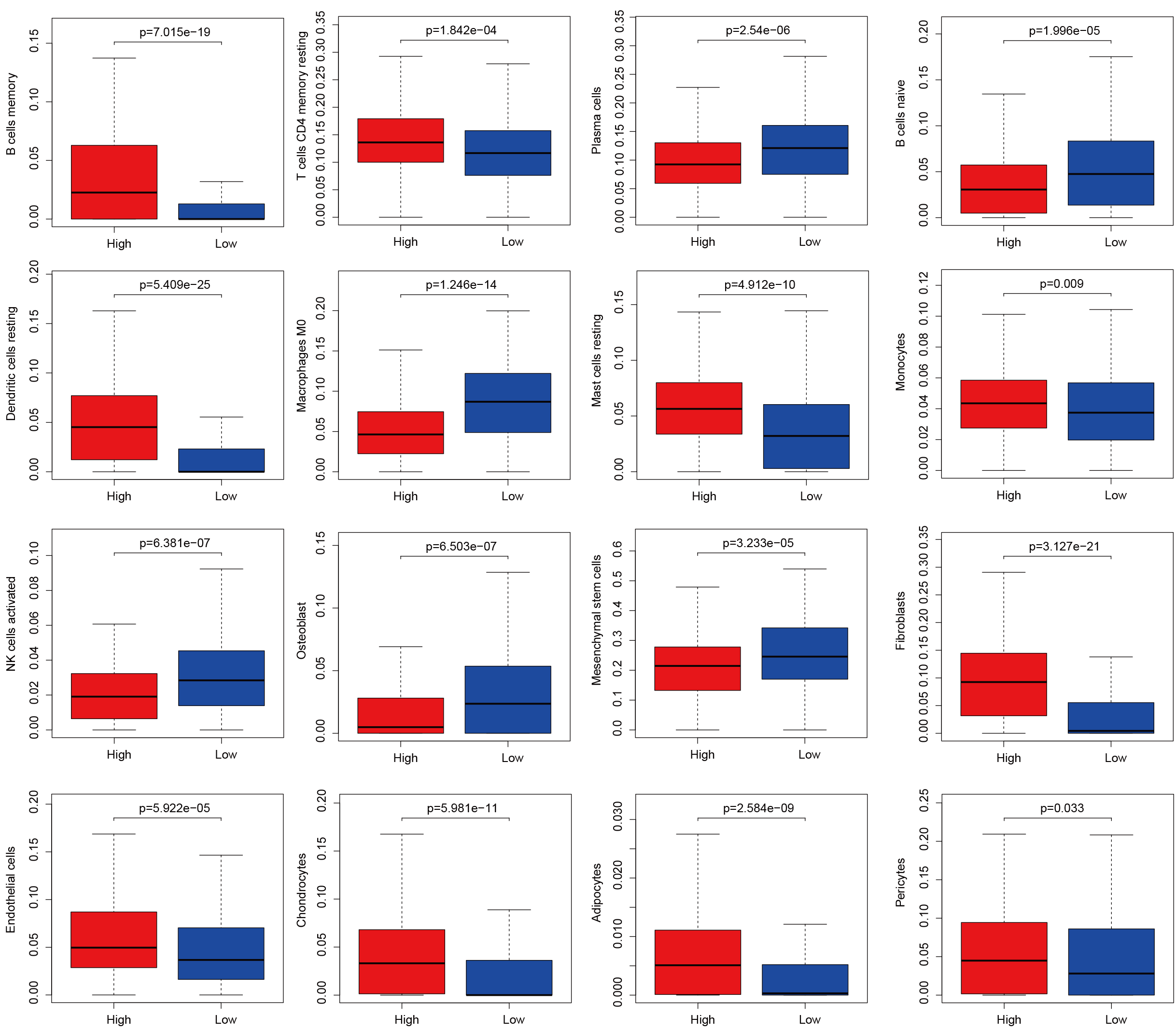

Supplement: Supplemental Information 3 [file peerj-08-9530-s003.png]
